# Malignant breast adenomyoepithelioma with diagnostic discordance: a case report and literature review

**DOI:** 10.3389/fonc.2026.1844137

**Published:** 2026-07-08

**Authors:** Jing-Jou Lo, Lu-Pei Pan, Ming-Feng Hou, Hidenobu Takahashi

**Affiliations:** 1 Division of Breast Oncology and Surgery, Department of Surgery, Kaohsiung Medical University Hospital, Kaohsiung Medical University, Kaohsiung, Taiwan; 2 Department of Pathology, Kaohsiung Medical University Hospital, Kaohsiung Medical University, Kaohsiung, Taiwan; 3 Kaohsiung Breast Cancer Prevention and Education Society, Kaohsiung, Taiwan

**Keywords:** aged, breast neoplasms, diagnostic errors, large-core needle biopsy, malignant adenomyoepithelioma, review literature

## Abstract

Malignant adenomyoepithelioma (MAME) of the breast is a rare biphasic neoplasm with potential for local recurrence and distant metastasis. Preoperative diagnosis is often difficult because core needle biopsy (CNB) may undersample the biphasic and malignant features of the tumor. We report an 84-year-old woman, among the oldest described patients, who presented with a newly palpable left breast nodule. Breast ultrasonography demonstrated an indistinct hypoechoic subareolar lesion, and mammography showed a corresponding focal asymmetry without suspicious calcifications. CNB suggested invasive carcinoma of no special type with a triple-negative immunophenotype. The patient underwent breast-conserving surgery (BCS) with sentinel lymph node biopsy (SLNB) and an intraoperative radiotherapy (IORT) boost. Final pathology confirmed grade 3 malignant MAME measuring 16 mm, with perineural invasion and a Ki-67 proliferation index of 20%. Immunohistochemistry confirmed a biphasic epithelial and myoepithelial nature. Surgical margins were negative, and sentinel lymph nodes were uninvolved. The patient subsequently received adjuvant capecitabine chemotherapy and supplemental whole-breast irradiation (WBI), with only grade 1 hand–foot syndrome, and showed no evidence of disease at 15 months of short-term follow-up. A pooled literature review showed substantial discordance between CNB and final pathology, supporting complete excision with comprehensive immunohistochemical evaluation. The principal contribution of this report is to highlight histologic-subtype discordance, in contrast to the malignancy-level discordance more commonly described, as a complementary diagnostic challenge in MAME. Across reported cases, negative-margin resection remained the cornerstone of management, lymph node involvement was uncommon, and late recurrence or distant metastasis could occur.

## 1 Introduction

MAME of the breast is a rare biphasic tumor. It is composed of admixed epithelial and myoepithelial elements, and malignancy may arise in either or both components. Histologic features that signal malignant behavior include marked cytologic atypia, an increased mitotic rate (commonly defined as ≥10 mitoses per 10 high-power fields), nuclear pleomorphism, necrosis, and an invasive growth pattern. These features have been associated with a documented potential for local recurrence and distant metastasis (1–4).

Population-based data are scarce. A U.S. National Cancer Database study captured 110 breast adenomyoepithelioma/myoepithelioma cases recorded as malignant invasive disease from 2004 to 2013 (5). Although most adenomyoepitheliomas are benign, malignant transformation has been reported in a minority of cases, with an estimated incidence of 15–25% in published series (6, 7). When metastasis occurs, available reports suggest a predominantly hematogenous pattern, whereas regional lymph node involvement is uncommon but has been described (1, 4).

Diagnosis relies on histopathologic assessment with immunohistochemistry to confirm the dual cell populations and identify malignant features. Clinically, patients may present with a painless breast mass. Radiologic findings are generally nonspecific, which limits preoperative discrimination from other breast lesions (1, 8, 9). Recent literature has proposed classification schemes based on histological patterns and the extent of invasion (2).

Given the risk of recurrence and metastasis, treatment typically prioritizes complete surgical excision with negative margins, achieved by wide local excision or mastectomy (4, 6, 9). SLNB can be considered when lymphatic spread is suspected (1, 9). The role of adjuvant systemic therapy and radiotherapy remains individualized, because standardized strategies are constrained by limited disease-specific evidence. Accordingly, prolonged surveillance is advocated to detect late relapse or distant metastasis (1, 4, 9).

In view of the rarity of breast MAME and the limited evidence guiding treatment, we report an 84-year-old woman with a pathologically confirmed breast MAME, managed with BCS, SLNB, an IORT boost with supplemental WBI, and adjuvant capecitabine chemotherapy. We also performed a structured literature review of cases with extractable individual patient-level data. The principal contribution of this report is to articulate *histologic-subtype discordance* — in our patient, CNB suggested invasive carcinoma of no special type with a triple-negative immunophenotype, whereas the excision specimen established MAME — as a distinct diagnostic challenge complementary to the *malignancy-level discordance* (CNB suggesting benign or non-malignant lesions) more commonly reported in the literature.

## 2 Case presentation

### 2.1 Patient information and clinical findings

An 84-year-old woman presented in October 2024 with a newly palpable left breast mass. Her medical history was notable for a left breast intraductal papilloma that had been surgically excised in 2019, without documented recurrence thereafter. She reported no family history of breast cancer. No relevant psychosocial history or hereditary cancer-related information was identified from the available records. Physical examination revealed a firm, approximately 1.5 cm palpable nodule in the left breast. No palpable axillary or supraclavicular lymphadenopathy was detected on physical examination.

### 2.2 Timeline

**Figure 1:**
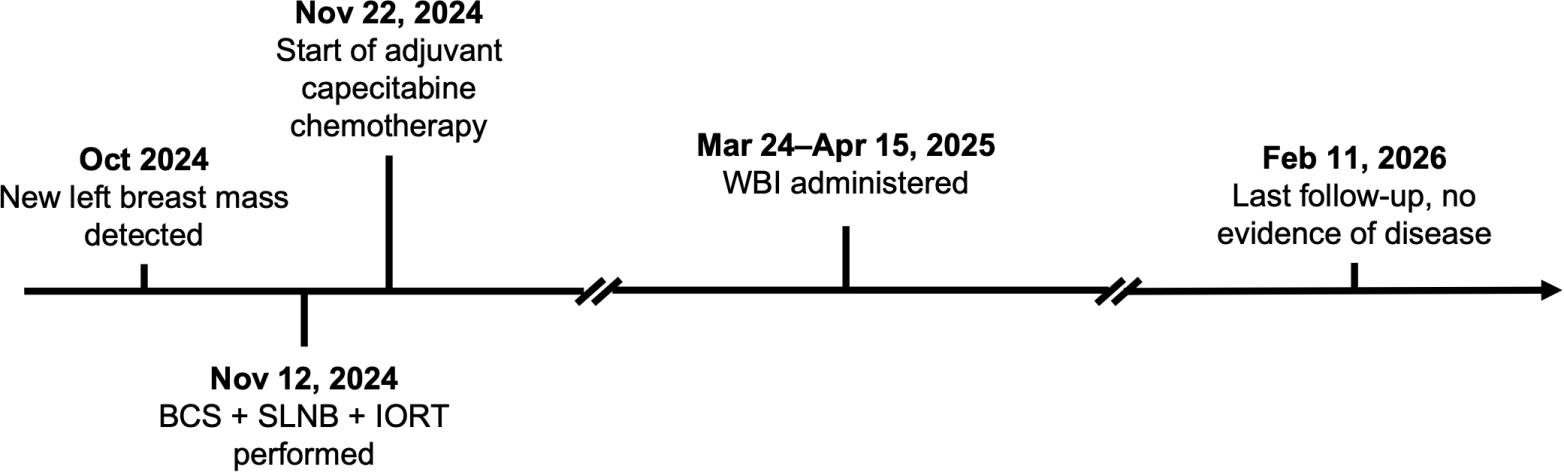
Clinical timeline. Chronological timeline of the patient’s clinical course from initial presentation to the most recent follow-up. In October 2024, a new mass was detected in the left breast. On 12 November 2024, the patient underwent breast-conserving surgery (BCS), sentinel lymph node biopsy (SLNB), and intraoperative radiotherapy (IORT). On 22 November 2024, adjuvant capecitabine chemotherapy was initiated; capecitabine was dose-reduced after the first cycle because of grade 1 hand–foot syndrome. From 24 March to 15 April 2025, supplemental whole-breast irradiation (WBI) was administered. At the last follow-up on 11 February 2026, there was no evidence of disease.

**Figure 1** summarizes the patient’s clinical course from initial presentation to the most recent follow-up; the timeline is referenced throughout the case narrative.

### 2.3 Diagnostic assessment

**Figure 2:**
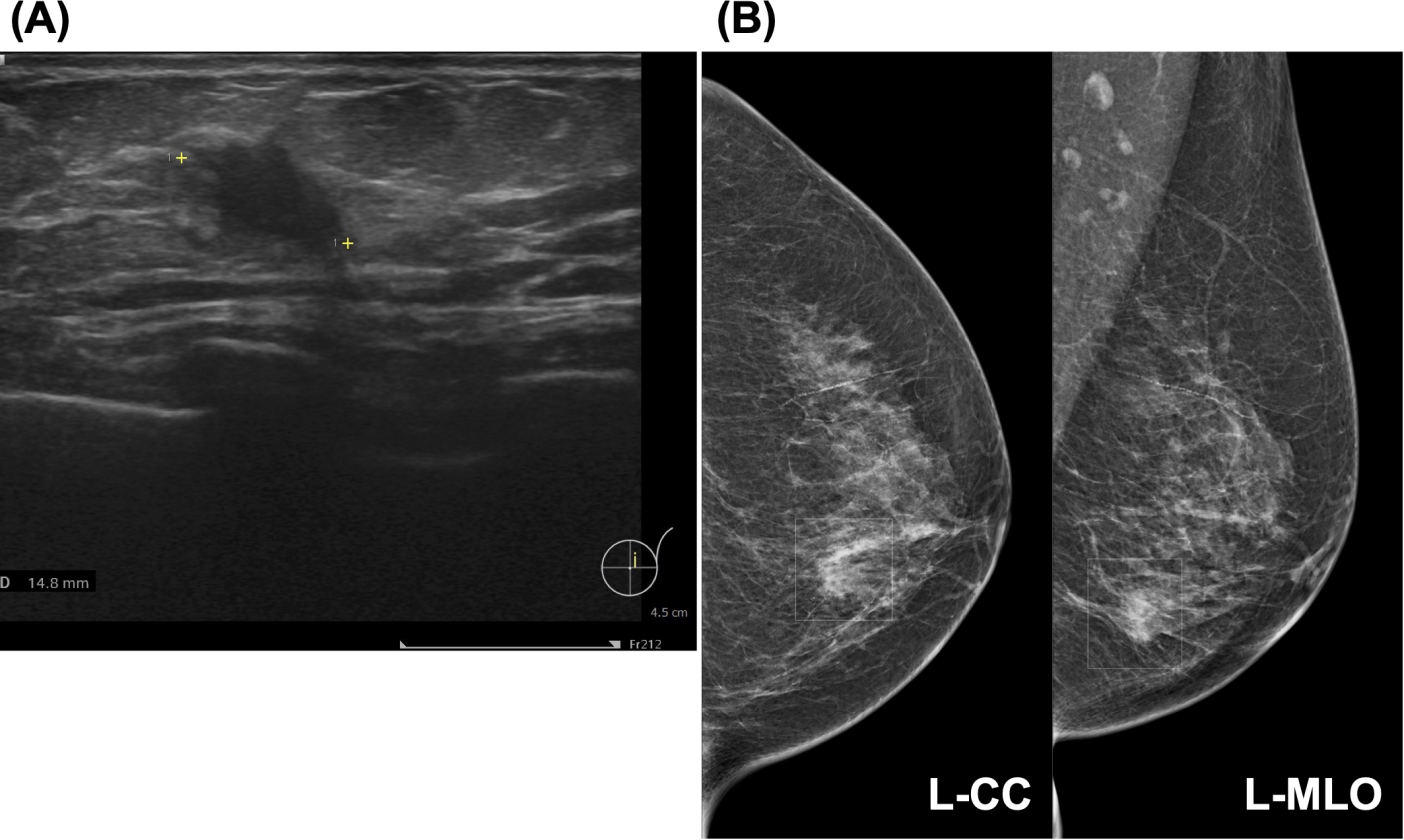
Preoperative breast imaging findings. **(A)** Breast ultrasonography of the left breast demonstrating an approximately 1.5 cm indistinct hypoechoic subareolar nodule, categorised as BI-RADS 4C. **(B)** Left breast mammography in craniocaudal (CC) and mediolateral oblique (MLO) views showing a developing asymmetry measuring approximately 1.5 cm in the lower inner quadrant without suspicious calcifications, categorised as BI-RADS 4C.

Breast ultrasonography (**Figure 2A**) demonstrated an approximately 1.5 cm indistinct hypoechoic lesion in the subareolar, classified as BI-RADS 4C. Mammography (**Figure 2B**) revealed a developing asymmetry in the lower inner quadrant, approximately 1.5 cm, without suspicious calcifications, classified as BI-RADS 4C. A core needle biopsy (CNB) of the breast mass revealed invasive carcinoma of no special type with a triple-negative profile (estrogen receptor (ER) negative, progesterone receptor (PR) negative, and human epidermal growth factor receptor 2 (HER2) negative) and low proliferative index (Ki-67 approximately 5%). Staging computed tomography and bone scan showed no distant metastasis. The preoperative impression was triple-negative invasive breast carcinoma, whereas the final diagnosis was established only after excision and comprehensive histopathologic and immunohistochemical evaluation.

### 2.4 Therapeutic intervention

**Figure 3:**
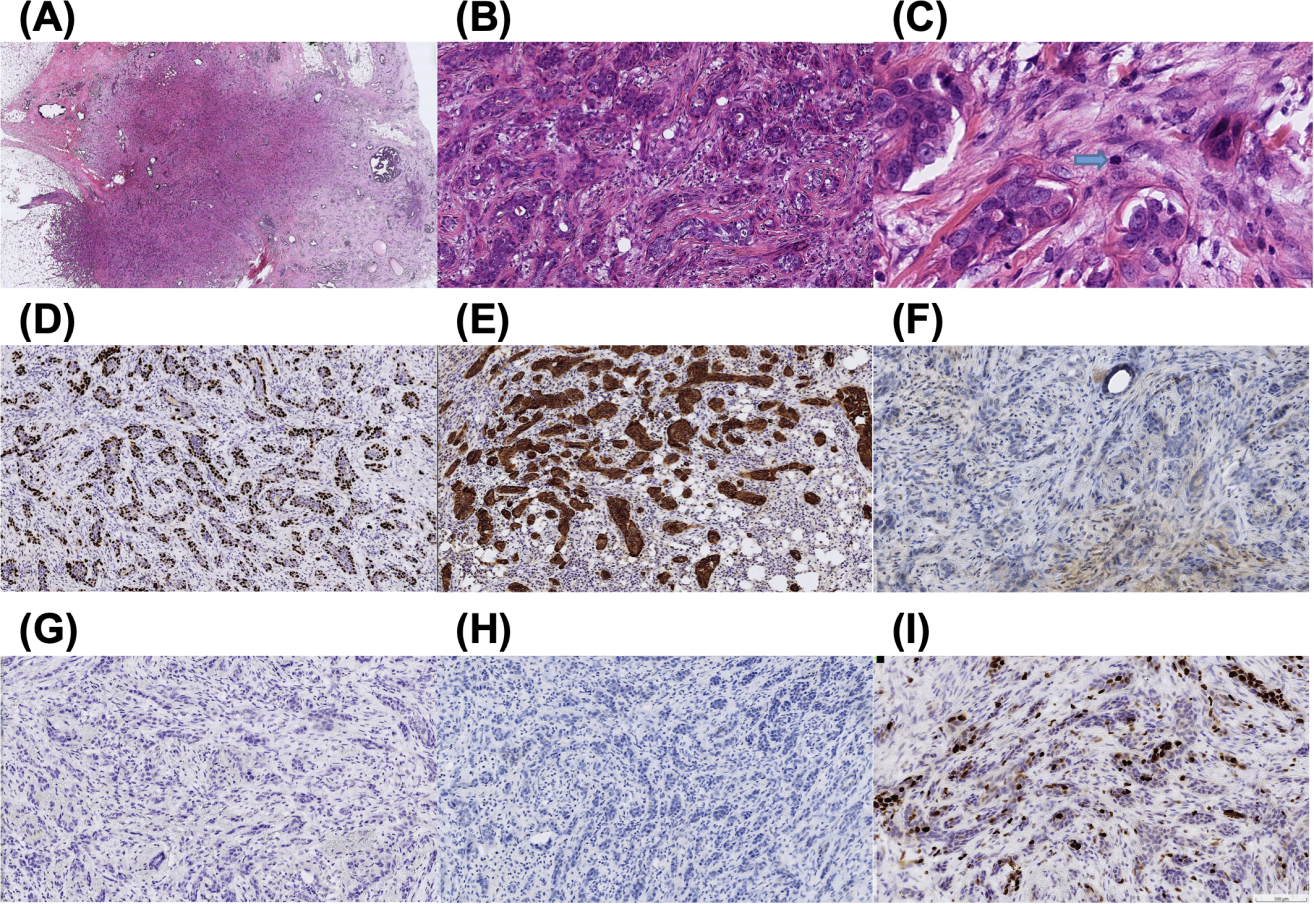
Histopathologic and immunohistochemical features of malignant adenomyoepithelioma. **(A)** Low-power haematoxylin and eosin view showing an infiltrative tumour border. **(B)** Haematoxylin and eosin staining showing biphasic proliferation of epithelial and myoepithelial cells in a collagenized stroma. **(C)** High-power haematoxylin and eosin view demonstrating cytologic atypia, prominent nucleoli, and increased mitotic activity, with 10 mitoses per 10 high-power fields; the blue arrow indicates a mitotic figure. **(D)** p63 immunostaining highlights the myoepithelial cells. **(E)** Cytokeratin 5/6 immunostaining highlights both epithelial and myoepithelial components. **(F)** Tumour cells are negative for estrogen receptor. **(G)** Tumour cells are negative for progesterone receptor. **(H)** Tumour cells are negative for human epidermal growth factor receptor 2. **(I)** The Ki-67 labelling index is approximately 20%.

The preoperative American Society of Anesthesiologists physical status was class III. The patient underwent left BCS with SLNB. Four sentinel lymph nodes were removed and all were negative (0/4). IORT was delivered intraoperatively to the tumor bed as a single 20 Gy boost. Postoperative histopathology confirmed the diagnosis of MAME, Nottingham grade 3 (score 8), with a 16 mm invasive component. Perineural invasion was present; no lymphovascular invasion or ductal carcinoma *in situ* was identified. The carcinoma demonstrated a biphasic morphology with distorted glandular structures embedded in a collagenized stroma and infiltrative borders. Cytologic atypia was marked, with enlarged pleomorphic nuclei and frequent mitoses. Immunohistochemical stains highlighted the dual cell populations: the tumor showed diffuse strong positivity for myoepithelial markers (p63, cytokeratin 5/6, calponin, smooth muscle myosin heavy chain) confirming the biphasic epithelial/myoepithelial differentiation, while ER and PR were 0% and HER2 was 0 (non-amplified). The Ki-67 proliferation index was approximately 20%. All surgical margins were negative. Representative histopathologic and immunohistochemical findings are shown in **Figure 3**. The pathologic stage was pT1c pN0(sn) cM0, prognostic stage IB, according to the 8th edition of the American Joint Committee on Cancer staging system. At the multidisciplinary breast cancer board, adjuvant systemic chemotherapy was recommended because of adverse clinicopathologic features, including high-grade histology and perineural invasion. After shared decision-making, the patient preferred an oral regimen, and capecitabine was selected at 500 mg three times daily for 14 days every 21 days. The dose was subsequently reduced to 500 mg twice daily because of grade 1 hand-foot syndrome. Supplemental WBI (40.05 Gy in 15 fractions) was delivered following the IORT boost.

### 2.5 Follow-up and outcomes

Treatment was well tolerated, with only grade 1 hand–foot syndrome and no other clinically relevant adverse events. At 15 months after surgery — a short-term timepoint relative to the 8–60 month range of distant metastases reported in our pooled analysis — the patient remained without clinical, radiologic, or biochemical evidence of local recurrence or distant metastasis. Follow-up was planned with reference to general breast cancer surveillance practice, with attention to late events given the documented potential for delayed recurrence in MAME. The present report therefore provides short-term feasibility and tolerability data, and continued long-term surveillance is required before durable conclusions can be drawn.

## 3 Literature review

### 3.1 Methods

We performed a structured, PRISMA-style literature review to identify published case reports and case series of MAME of the breast with extractable patient-level data.

#### 3.1.1 Databases and date range

PubMed and Google Scholar were searched for studies published between 1 January 2010 and 31 December 2024. Reference lists of included articles were also screened for additional cases.

#### 3.1.2 Search strategy

The PubMed strategy combined the terms (“malignant adenomyoepithelioma” OR “adenomyoepithelial carcinoma” OR “adenomyoepithelioma with carcinoma”) AND (“breast”) AND (“case report” OR “case series” OR “clinicopathologic”). Filters: English language, human studies, publication date 2010-01–01 to 2024-12-31. Google Scholar was searched with the same key terms to capture additional indexed and grey literature.

#### 3.1.3 Eligibility criteria

Inclusion: original case reports or case series of histologically confirmed breast MAME, published in English, with extractable clinical and pathologic data at the patient level. Exclusion: benign adenomyoepithelioma without documented malignant transformation, non-breast adenomyoepithelioma, conference abstracts without extractable case-level data, and duplicate publications of the same case.

#### 3.1.4 Study selection and data extraction

Titles and abstracts were screened, followed by full-text assessment of potentially eligible articles. Two authors independently extracted data on patient demographics, tumor size, hormone-receptor and HER2 status, CNB diagnosis (with malignancy-level concordance assessed against final excision pathology), surgical and axillary management, adjuvant therapy, follow-up duration, and oncologic outcomes (local recurrence, distant metastasis, sites of metastasis, and status at last follow-up). Discrepancies were resolved by consensus. The flow of records through identification, screening, eligibility assessment, and inclusion is summarized in **Supplementary Figure 1** (PRISMA-style flow diagram). Ten publications met the inclusion criteria, contributing 27 cases (4, 10, 11–13, 14–18); together with the present case, 28 cases were analyzed.

#### 3.1.5 Statistical and interpretive approach

Pooled summary statistics are descriptive. Where appropriate, exact 95% confidence intervals (CI) for proportions are reported. Given the small sample size, heterogeneity in reporting (variable case definitions, follow-up duration, and immunohistochemistry panels), and likely publication bias, all pooled estimates are interpreted cautiously and should not be regarded as meta-analytic.

### 3.2 Results

Across the published cases summarized in **Table 1**, 27 cases were retrieved from the literature; together with the present case, 28 cases were available for comparative analysis. Detailed case-level data are provided in **Supplementary Table 1**. Patient age at diagnosis had a median of 58 (34–84) years (reported n=15). Tumor size had a median of 1.6 (0.4–7.0) cm (reported n=25). ER, PR, and HER2 status were reported in 23, 22, and 20 cases, with positivity in 5/23 (21.7%), 5/22 (22.7%), and 3/20 (15.0%) cases, respectively.

**Table 1:** Summary of pooled clinicopathologic characteristics, treatments, and outcomes in published cases of breast malignant adenomyoepithelioma.

Measure	Value	Available data, n
Number of cases	28	28
Median age (range), years	58 (34–84)	15
Median tumor size (range), cm	1.6 (0.4–7.0)	25
Estrogen receptor positive	5/23 (21.7%)	23
Progesterone receptor positive	5/22 (22.7%)	22
Human epidermal growth factor receptor 2 positive	3/20 (15.0%)	20
Core needle biopsy–final pathology discordance, malignancy level	7/23 (30.4%; 95% CI, 13.2–52.9)	23
Lumpectomy	19/28 (67.9%)	28
Mastectomy	9/28 (32.1%)	28
Sentinel lymph node biopsy	14/28 (50.0%)	28
Axillary lymph node dissection	2/28 (7.1%)	28
Axillary lymph node metastasis	1/16 (6.2%)	16
Chemotherapy	9/27 (33.3%)	27
Radiotherapy	14/27 (51.9%)	27
Endocrine therapy	6/27 (22.2%)	27
Follow-up, median (range), months	34 (4–162)	24
Local recurrence	2/28 (7.1%; 95% CI, 0.9–23.5)	28
Time to local recurrence, median (range), months	25 (10–40)	2
Distant metastasis	6/28 (21.4%; 95% CI, 8.3–41.0)	28
Most common distant metastatic site: Lung	4/6 (66.7%)	6
Time to distant metastasis, median (range), months	15 (8–60)	6
Any recurrence	6/28 (21.4%; 95% CI, 8.3–41.0)	28
Time to first recurrence, median (range), months	14 (8–60)	6

Cases with available data, n indicates the number of evaluable cases for each variable. 95% confidence intervals (CI) are shown where applicable.

Preoperative CNB results were discordant with the final malignant pathology in 7/23 (30.4%; 95% CI 13.2–52.9) cases, in which CNB suggested non-malignant lesions. The discordant CNB diagnostic labels included intraductal papilloma, eccrine spiradenoma, papillary neoplasm, sclerosing adenosis, and breast adenoma. Our case represents a different, complementary pattern — histologic-subtype discordance — in which CNB correctly indicated malignancy but misclassified the histologic subtype.

Surgical management consisted of lumpectomy in 19/28 (67.9%) and mastectomy in 9/28 (32.1%). SLNB was performed in 14/28 (50.0%) cases and axillary lymph node dissection (ALND) in 2/28 (7.1%) cases. Axillary lymph node metastasis was reported in 1/16 (6.2%) cases with documented node status. Adjuvant therapy reporting was available for n=27: chemotherapy was administered in 9/27 (33.3%), radiotherapy in 14/27 (51.9%), and endocrine therapy in 6/27 (22.2%).

Follow-up duration had a median of 34 (4–162) months (reported n=24). **Supplementary Figure 2** visualizes individual follow-up timelines and the timing of local recurrence and distant metastasis, with status at last follow-up. Local recurrence occurred in 2/28 (7.1%; 95% CI 0.9–23.5) cases, with a median time to local recurrence of 25 (10–40) months (reported n=2). Distant metastasis occurred in 6/28 (21.4%; 95% CI 8.3–41.0) cases, with a median time to distant metastasis of 15 (8–60) months (reported n=6). Any recurrence (local recurrence and/or distant metastasis) was reported in 6/28 (21.4%; 95% CI 8.3–41.0) cases, with a median time to first recurrence of 14 (8–60) months (reported n=6). Among cases with reported sites of distant metastasis, the lung was the most common, occurring in 4/6 cases (66.7%). Given the small numbers, wide CIs, and heterogeneity, these descriptive estimates should be interpreted cautiously.

## 4 Discussion

MAME of the breast is a rare biphasic neoplasm with limited evidence to guide management. Although most reported cases occur in middle-aged women, population-level data remain sparse and largely derive from retrospective cohorts and pooled case reports (1, 5, 6, 10). In our dataset, the median reported age was 58 years (34–84; n=15), and our 84-year-old patient represents the upper extreme of reported age. This underscores the geriatric-specific tradeoffs between oncologic control and tolerability that informed every treatment decision.

Establishing a preoperative diagnosis of MAME is difficult. CNB findings are often discordant in the setting of architectural and cytologic heterogeneity, particularly when sampling captures only a small portion of the lesion. In such limited specimens, the biphasic composition (epithelial and myoepithelial components) and the malignant features (cytologic atypia, high mitotic rate, invasion) may be missed, resulting in underdiagnosis or misclassification (3, 19, 20). It is important to distinguish two patterns of discordance: *malignancy-level discordance*, in which CNB suggests a benign or non-malignant lesion (the dominant pattern in the literature, observed in 7/23 cases (30.4%; 95% CI 13.2–52.9) of our pooled dataset and in 6/15 cases (40%) in a previous series) (10), and *histologic-subtype discordance*, in which CNB correctly identifies malignancy but misclassifies the histologic subtype. Our case illustrates the latter: CNB suggested invasive carcinoma of no special type with a triple-negative immunoprofile, whereas the excision specimen established MAME, supported by myoepithelial marker expression (p63, smooth muscle myosin heavy chain, calponin) and basal cytokeratin staining (cytokeratin 5/6).

Accurate diagnosis relies on integrating histologic evaluation with immunohistochemistry. Recommended panels include epithelial markers — cytokeratin and epithelial membrane antigen — together with myoepithelial markers including p63, S-100, smooth muscle actin, calponin, and CD10 (1, 3, 20, 21). Diagnostic criteria for malignancy in MAME include marked cytologic atypia, brisk mitotic activity (commonly defined as ≥10 mitoses per 10 high-power fields), tumor necrosis, an infiltrative growth pattern, and tumor size ≥2 cm; one or more of these features in either the epithelial or myoepithelial component supports a malignant designation (2, 20, 21). Two principal differential diagnoses warrant emphasis. Metaplastic carcinoma is a predominantly monophasic carcinoma with squamous, spindle-cell, or heterologous mesenchymal differentiation; it typically lacks the dual epithelial/myoepithelial architecture and the dual-marker immunoprofile that characterizes MAME. Myoepithelial carcinoma is a monophasic malignant myoepithelial proliferation without an epithelial component; the absence of bilineage differentiation distinguishes it from MAME. Recognizing these entities is important because management considerations and prognostic implications may differ.

Histologically, MAME of the breast is characterized by marked cytologic atypia and brisk mitotic activity, together with local invasion into adjacent breast tissue, pleomorphism, and necrosis involving one or both cellular components (2, 20, 21). Factors linked to local recurrence or distant metastasis include a high mitotic rate, marked cytologic atypia, an infiltrative growth pattern, tumor size ≥2 cm, and incomplete excision or positive surgical margins (4, 20, 21). Our patient’s tumor exhibited several adverse features — Nottingham grade 3, approximately 10 mitoses per 10 high-power fields, perineural invasion, and a Ki-67 proliferation index of approximately 20% — despite a relatively small invasive component (16 mm) and complete excision with negative margins.

By immunohistochemistry, ER, PR, and HER2 expression in MAME is typically absent in both the epithelial and myoepithelial components, resulting in a frequent “triple-negative” immunophenotype (5, 22). An important caveat is that this profile likely reflects myoepithelial differentiation — myoepithelial cells are typically ER/PR/HER2-negative — rather than basal-like or conventional triple-negative breast cancer (TNBC) biology (22–24). Consequently, MAME with a triple-negative immunoprofile should not be assumed to be biologically equivalent to conventional TNBC. To date, available data do not suggest a consistent association between receptor status and clinical outcomes in MAME (5, 22). In our pooled dataset, 13/20 (65.0%) cases with complete receptor reporting were ER/PR/HER2-negative.

Surgical management for MAME of the breast consists of complete resection with negative margins. Wide local excision is typically favored, whereas mastectomy may be considered for larger or more aggressive tumors or when BCS cannot achieve negative margins (4, 6, 8, 9, 20). Securing clear margins is essential, as incomplete resection has been associated with an increased risk of local recurrence and possible metastasis (4, 6, 20). No specific margin width has been established by consensus; however, published reports uniformly highlight the importance of histologically negative margins (6, 20). In our dataset, lumpectomy was common (19/28; 67.9%), with mastectomy performed in 9/28 cases (32.1%). Local recurrence was observed in 2/28 cases (7.1%; 95% CI 0.9–23.5), with a median time to local recurrence of 25 months (10–40), supporting the concept that local failure can occur beyond the early postoperative window.

Available evidence suggests that lymph node metastasis in MAME is uncommon (1, 5, 6, 8, 9). Hematogenous dissemination is thought to be the dominant metastatic pathway. When distant metastasis occurs, the lung and brain are the most frequently reported sites, a distribution consistent with hematogenous spread (4, 21, 25). Nevertheless, isolated cases with axillary involvement have been described, including reports of nodal metastasis in histologically malignant disease, supporting a pragmatic rationale for SLNB in invasive or high-grade tumors (1, 8, 9). Routine ALND appears difficult to justify given the low reported positivity and morbidity profile, and omission of axillary surgery may be reasonable in clearly benign or low-grade lesions without invasive features, although data remain limited (5). In our dataset, SLNB was performed in 14/28 cases (50.0%) and ALND in 2/28 cases (7.1%); nodal positivity was reported in only 1/16 cases (6.2%) with documented node status.

Adjuvant therapy in MAME is not standardized, and its use varies widely. In a national cohort analysis (n=110), adjuvant treatments were used in a minority of patients — radiotherapy in 40/110 cases (36.4%), chemotherapy in 29/110 cases (26.4%), and endocrine therapy in 9/110 cases (8.2%) — and at a median follow-up of 52 months, 5-year overall survival was 74.4%, with no clear association between adjuvant treatment and overall survival (5). These findings highlight the uncertainty and the need for individualized decision-making. In our dataset (n=27 with reporting), radiotherapy was administered in 14/27 cases (51.9%), chemotherapy in 9/27 cases (33.3%), and endocrine therapy in 6/27 cases (22.2%), reflecting clinician concern for local control and metastatic risk in selected patients.

There is no MAME-specific evidence for radiotherapy. Strategies are extrapolated from the early-stage breast cancer literature, where WBI after BCS reduces ipsilateral breast events and, in some analyses, modestly improves breast cancer outcomes (26, 27). A tumor-bed boost further improves local control in higher-risk settings, and IORT delivered as a boost combined with WBI has shown acceptable oncologic outcomes in broader breast cancer populations (28–32). On this evidence-extrapolated basis, our patient received an IORT boost with supplemental WBI as an individualized strategy intended to balance local control against treatment burden in advanced age. We acknowledge that this approach is not validated for MAME specifically, and the absence of disease-specific radiotherapy evidence is an explicit limitation.

For systemic therapy, MAME-specific evidence is sparse. Reported regimens are heterogeneous and largely limited to case reports or small series, often employing anthracycline- and/or taxane-based chemotherapy in selected high-risk presentations (1, 10–13). No direct evidence supports adjuvant capecitabine specifically for breast MAME. In our patient, oral capecitabine was selected as an individualized decision in the absence of disease-specific evidence, based on (i) the ER/PR/HER2-negative immunophenotype frequently observed in MAME, (ii) cautious extrapolation from CREATE-X, in which adjuvant capecitabine improved disease-free and overall survival in HER2-negative patients with residual invasive disease after neoadjuvant chemotherapy (including the TNBC subgroup) (33, 34), and from SYSUCC-001, in which extended (metronomic) capecitabine after standard therapy improved disease-free survival in early TNBC (35, 36), (iii) the patient’s preference for an oral regimen, and (iv) tolerability considerations in advanced age. We re-emphasise that “triple-negative by IHC” in biphasic tumors may not be biologically equivalent to conventional TNBC, and therefore extrapolation must remain cautious.

Outcome estimates for MAME remain imprecise because of small numbers, heterogeneous reporting, and publication bias. The lung and brain are the most frequently reported distant metastatic sites (4, 21, 37), and metastasis typically occurs between 6 months and several years after the initial diagnosis, often preceded by repeated local recurrences (1, 21, 38). In our dataset (n=28), local recurrence occurred in 2/28 cases (7.1%; 95% CI 0.9–23.5) with a median time to local recurrence of 25 months (range 10–40), and distant metastasis occurred in 6/28 cases (21.4%; 95% CI 8.3–41.0) with a median time to distant metastasis of 15 months (range 8–60). The lung was the most common metastatic site (4/6, 66.7%). Our patient remained without evidence of disease at 15 months — a timepoint that coincides with the early end of the documented metastasis window — so the present report contributes short-term feasibility and tolerability data rather than long-term efficacy. Continued long-term surveillance is essential before durable conclusions can be drawn.

Owing to its rarity, post-treatment surveillance for MAME is largely extrapolated from conventional breast cancer follow-up guidelines, with closer monitoring considered in cases with adverse pathology or prior recurrence (3, 5, 8, 20, 37).

Similar diagnostic challenges — atypical presentation, reliance on excisional pathology for definitive diagnosis, and management decisions extrapolated from conventional breast cancer protocols — have been described in other rare breast cancer presentations outside the usual anatomic or histologic context, such as invasive ductal carcinoma arising in axillary ectopic breast tissue (39). Together with our case, these reports highlight that diagnostic complexity in rare breast malignancies is a recurring theme that warrants pre-defined multidisciplinary pathways and prolonged surveillance.

### 4.1 Strengths and limitations

A strength of this case is the achievement of negative-margin breast-conserving treatment with individualized multimodality therapy in an elderly patient, and the inclusion of a structured PRISMA-style literature review with explicit subtype-level discordance analysis. However, the short follow-up duration and the absence of disease-specific evidence for adjuvant systemic therapy limit conclusions regarding long-term oncologic benefit. The pooled case-level literature is constrained by publication and selection bias, heterogeneous and incomplete reporting, and the small number of recurrence and metastatic events, which preclude time-to-event inference. Accordingly, the effects of radiotherapy and systemic therapy, including capecitabine, remain uncertain. Longer follow-up of the present case is warranted, given the potential for late recurrence or distant metastasis.

## 5 Conclusion

MAME of the breast is a rare biphasic neoplasm with heterogeneous behavior and limited evidence to guide standardized management. Our pooled analysis highlights substantial preoperative diagnostic uncertainty: malignancy-level discordance between CNB and final pathology was observed in 30.4% of cases. Our case adds histologic-subtype discordance — in which CNB correctly identifies malignancy but misclassifies the histologic subtype — as a complementary diagnostic challenge. Together, these patterns underscore the need for complete excision and comprehensive immunohistochemical evaluation when an epithelial–myoepithelial lesion is suspected. Across reported cases, definitive surgical resection with negative margins remains the cornerstone of treatment. Lymph node involvement appears uncommon, so SLNB may be a pragmatic staging approach in invasive or high-grade presentations. An ER/PR/HER2-negative immunophenotype is frequently reported but should be interpreted as an immunophenotypic descriptor of biphasic tumors rather than a marker of conventional TNBC biology.

In the present 84-year-old patient, BCS with negative margins, SLNB (0/4), an IORT boost followed by supplemental WBI, and individualized adjuvant oral capecitabine — selected in the context of high-grade features and patient preference — were feasible and well tolerated, with no evidence of disease at 15 months of short-term follow-up. Given the documented potential for distant metastasis occurring months to years after diagnosis, long-term surveillance remains warranted before any durable efficacy conclusion can be drawn. Larger multicenter registries with standardized pathology review and uniform outcome reporting are needed to refine prognostication and clarify the role of adjuvant systemic therapy in MAME.

## 6 Patient perspective

From the patient’s perspective, the breast-conserving approach was reassuring, and the oral chemotherapy regimen was acceptable because it allowed her to maintain her independence and remain at home with her family.

## Data Availability

The original contributions presented in the study are included in the article/**Supplementary Material**. Further inquiries can be directed to the corresponding author.

## Supplementary material

Supplementary Figure 1 Individual follow-up timelines, with timing of local recurrence and distant metastasis, and status at last follow-up.

Supplementary Figure 2 PRISMA-style flow diagram of the literature search and study selection.

Supplementary Table 1 Detailed case-level data extracted from the included publications.
