# Supplementary material for: Malignant breast adenomyoepithelioma with diagnostic discordance: a case report and literature review
Source: Front Oncol. 2026 Jul 8;16:1844137. doi: 10.3389/fonc.2026.1844137 (PMC13388142; doi:10.3389/fonc.2026.1844137)
Supplement: Supplementary Figure 1 — Individual follow-up timelines, with timing of local recurrence and distant metastasis, and status at last follow-up. [file Image1.pdf]

Supplementary Figure 1. PRISMA-style flow diagram of the literature search

Breast malignant adenomyoepithelioma — case report with pooled literature analysis

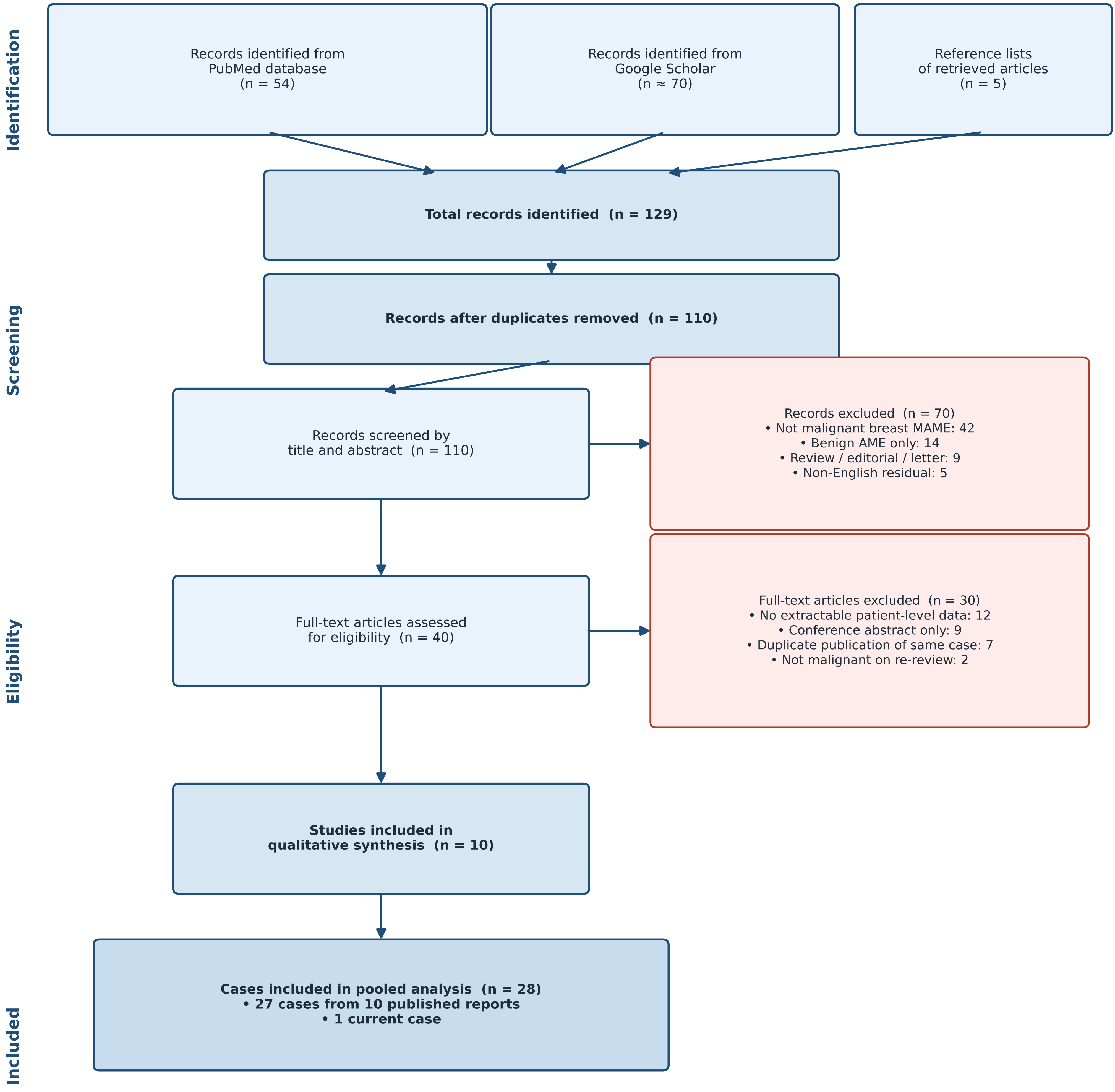

MAME = malignant adenomyoepithelioma of the breast.
Search dates: 1 January 2010 – 31 December 2024. Language: English.
PubMed Boolean string: ("malignant adenomyoepithelioma" OR "adenomyoepithelial carcinoma" OR "adenomyoepithelioma with carcinoma") AND breast AND English[lang]. PubMed yield re-verified 2026-05-07.
Google Scholar yield reflects the operator's screening at the time of search and is reported as an approximate value because Google Scholar does not return a stable hit count across queries.
