# Supplementary figures and images for: Malignant breast adenomyoepithelioma with diagnostic discordance: a case report and literature review
Source: Front Oncol. 2026 Jul 8;16:1844137. doi: 10.3389/fonc.2026.1844137 (PMC13388142; doi:10.3389/fonc.2026.1844137)

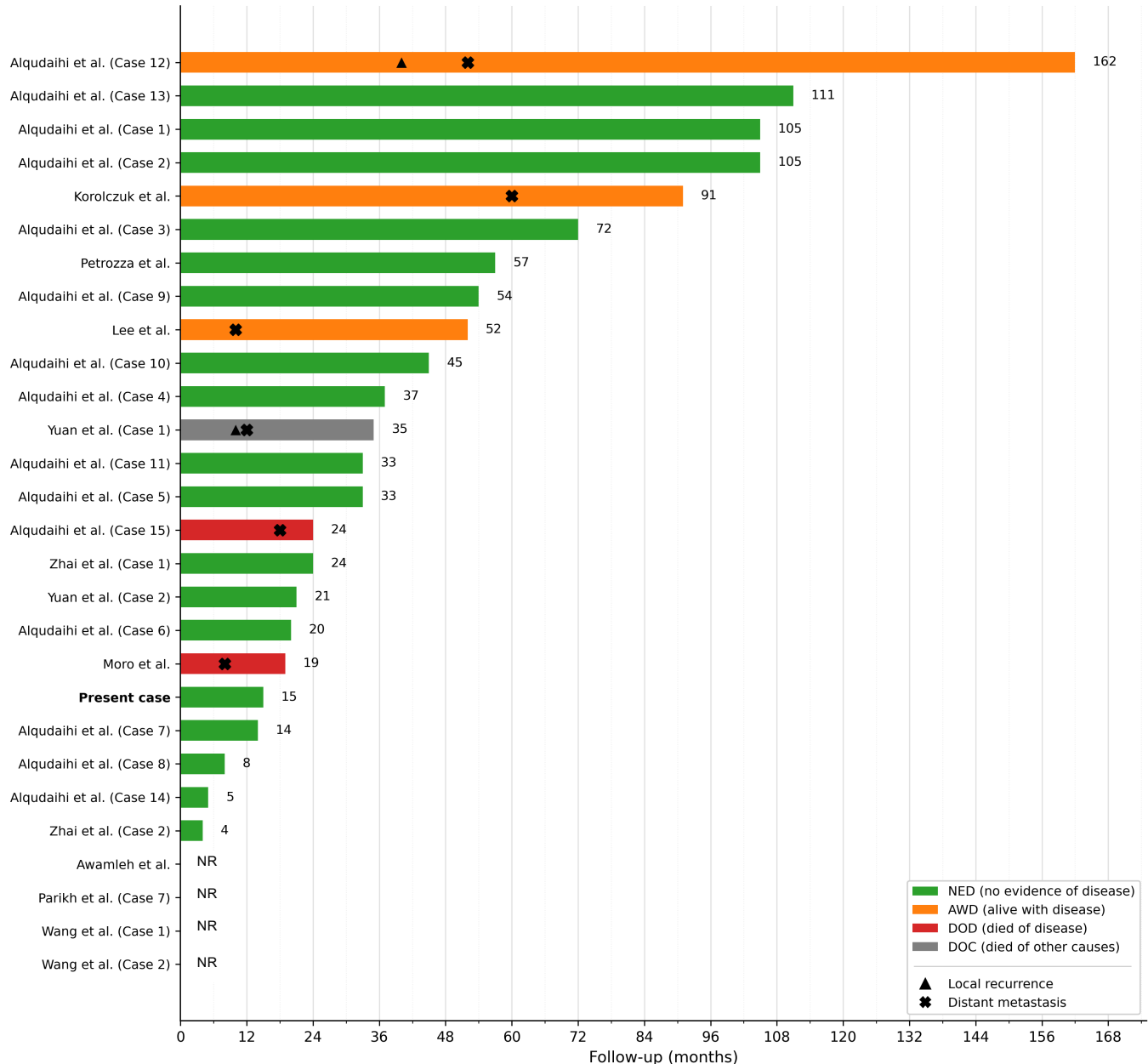

Supplement: Supplementary Figure 2 — PRISMA-style flow diagram of the literature search and study selection. [file Image2.pdf]
