# Supplementary material for: Malignant breast adenomyoepithelioma with diagnostic discordance: a case report and literature review
Source: Front Oncol. 2026 Jul 8;16:1844137. doi: 10.3389/fonc.2026.1844137 (PMC13388142; doi:10.3389/fonc.2026.1844137)
Supplement: Supplementary Table 1 — Detailed case-level data extracted from the included publications. [file Table1.docx]

Supplementary Table 1. Detailed case-level data from the literature review of published breast malignant adenomyoepithelioma cases.

| **Author (Year) / Case** | **Age (yr)** | **CNB Diagnosis** | **Final Pathologic Diagnosis** | **Surgery** | **Tumor Size (cm)** | **Nodes positive / examined** | **IHC (ER/PR/HER2)** | **CT** | **RT** | **ET** | **Follow-up (mo)** | **Time to LR (mo)** | **Time to DM (mo)** | **DM Site(s)** | **Status at Last Follow-up** |
| --- | --- | --- | --- | --- | --- | --- | --- | --- | --- | --- | --- | --- | --- | --- | --- |
| Present case | 84 | NST | MAME | BCS + SLNB | 1.6 | 0/4 | -/-/- | Yes | Yes | No | 15 |  |  |  | NED |
| Alqudaihi et al. (2022)(10) / Case 1 | NR | IDP | MAME | WLE | 1.3 | NA | +/+/- | No | Yes | Yes | 105 |  |  |  | NED |
| Alqudaihi et al. (2022)(10) / Case 2 | NR | NST | MAME with DCIS | BCS + SLNB | 1.5 | NR | -/-/- | No | Yes | No | 105 |  |  |  | NED |
| Alqudaihi et al. (2022)(10) / Case 3 | NR | AME tumor | MAME | TM + SLNB | 2.5 | NR | -/-/- | Yes | No | No | 72 |  |  |  | NED |
| Alqudaihi et al. (2022)(10) / Case 4 | NR | MAME | MAME | WLE + SLNB | 1.6 | NR | -/-/- | Yes | Yes | No | 37 |  |  |  | NED |
| Alqudaihi et al. (2022)(10) / Case 5 | NR | AME neoplasm | MAME | WLE | 1.0 | NA | NR/NR/NR | No | Yes | No | 33 |  |  |  | NED |
| Alqudaihi et al. (2022)(10) / Case 6 | NR | Eccrine spiradenoma | MAME | WLE | 2.8 | NA | -/-/- | No | No | No | 20 |  |  |  | NED |
| Alqudaihi et al. (2022)(10) / Case 7 | NR | Papillary neoplasm | MAME | WLE + SLNB | 0.8 | NR | +/+/- | No | Yes | Yes | 14 |  |  |  | NED |
| Alqudaihi et al. (2022)(10) / Case 8 | NR | MAME | MAME | WLE | 1.5 | NA | +/+/- | No | Yes | Yes | 8 |  |  |  | NED |
| Alqudaihi et al. (2022)(10) / Case 9 | NR | NST | MAME | BCS + SLNB | 0.4 | NR | -/-/+ | No | Yes | No | 54 |  |  |  | NED |
| Alqudaihi et al. (2022)(10) / Case 10 | NR | Papillary neoplasm | MAME | WLE | 1.3 | NA | +/-/- | No | Yes | Yes | 45 |  |  |  | NED |
| Alqudaihi et al. (2022)(10) / Case 11 | NR | AME neoplasm | MAME | WLE | 1.0 | NA | NR/NR/NR | No | Yes | No | 33 |  |  |  | NED |
| Alqudaihi et al. (2022)(10) / Case 12 | 65 | AME neoplasm | MAME | WLE | 7.0 | NA | +/+/NR | No | Yes | No | 162 | 40 | 52 | Lung | AWD |
| Alqudaihi et al. (2022)(10) / Case 13 | NR | Sclerosing adenosis | MAME | WLE | 1.6 | NA | NR/NR/NR | No | No | No | 111 |  |  |  | NED |
| Alqudaihi et al. (2022)(10) / Case 14 | NR | Papillary neoplasm | MAME | WLE | 0.8 | NA | NR/NR/NR | No | No | No | 5 |  |  |  | NED |
| Alqudaihi et al. (2022)(10) / Case 15 | 34 | NST | MAME | TM + SLNB | 1.7 | NR | -/-/- | Yes | No | Yes | 24 |  | 18 | Lung | DOD |
| Wang et al. (2022)(25) / Case 1 | 34 | Malignant epithelial tumor | MAME | BCS + SLNB | 2.5 | 0/4 | -/-/- | Yes | Yes | No | NR |  |  |  | NR |
| Wang et al. (2022)(25) / Case 2 | 45 | Not performed | MAME | BCS + SLNB | 2.2 | 0/5 | -/-/- | Yes | Yes | No | NR |  |  |  | NR |
| Parikh et al. (2021)(35) / Case 7 | 61 | Atypical epithelial-myoepithelial neoplasm | MAME | BCS | 1.5 | NA | -/-/- | No | No | No | NR |  |  |  | NR |
| Zhai et al. (2021)(26) / Case 1 | 46 | Breast adenoma composed of proliferating glandular ducts and a small amount of fibrous tissue. | MAME | WLE → TM + SLNB | 3.0 | 0/3 | -/-/- | Yes | No | No | 24 |  |  |  | NED |
| Zhai et al. (2021)(26) / Case 2 | 58 | DCIS | MAME | TM + SLNB | 4.5 | 0/5 | -/-/- | Yes | No | No | 4 |  |  |  | NED |
| Moro et al. (2020)(36) | 64 | NR | MAME | TM + ALND | NR | 0/26 | NR/NR/NR | No | No | No | 19 |  | 8 | Lung | DOD |
| Yuan et al. (2017)(27) / Case 1 | 58 | No definitive diagnosis | MAME | WLE | 3.0 | NA | -/+/+ | No | No | No | 35 | 10 | 12 | Bone | DOC |
| Yuan et al. (2017)(27) / Case 2 | 51 | Ductal epithelial and myoepithelial proliferation; focal atypical ductal hyperplasia. | MAME | WLE → TM + SLNB | 1.5 | 0/5 | -/-/+ | Yes | No | Yes | 21 |  |  |  | NED |
| Korolczuk et al. (2016)(37) | 56 | NR | MAME | TM | NR | NA | -/-/NR | No | No | No | 91 |  | 60 | Lung | AWD |
| Lee et al. (2015)(38) | 51 | NST | MAME | TM + ALND | NR | NR | -/-/- | No | No | No | 52 |  | 10 | Liver, Pleura, Abdominal wall | AWD |
| Petrozza et al. (2013)(4) | 60 | Not performed | MAME | BCS + SLNB | 2.4 | NR | -/-/- | No | Yes | No | 57 |  |  |  | NED |
| Awamleh et al. (2012)(39) | 63 | IDP with atypical hyperplasia | MAME | WLE → TM + SLNB | 7.0 | 1/2 | -/NR/NR | NR | NR | NR | NR |  |  |  | NR |

Abbreviations: ALND, axillary lymph node dissection; AME, adenomyoepithelioma; AWD, alive with disease; BCS, breast-conserving surgery; CNB, core needle biopsy; CT, chemotherapy; DCIS, ductal carcinoma in situ; DM, distant metastasis; DOC, dead of other causes; DOD, dead of disease; ER, estrogen receptor; ET, endocrine therapy; HER2, human epidermal growth factor receptor 2; IDP, intraductal papilloma; IHC, immunohistochemistry; LR, local recurrence; MAME, malignant adenomyoepithelioma; mo, months; NA, not applicable; NED, no evidence of disease; NR, not reported; NST, invasive carcinoma of no special type; PR, progesterone receptor; RT, radiotherapy; SLNB, sentinel lymph node biopsy; TM, total mastectomy; WLE, wide local excision; yr, years.
